# Mapping and analysis of a novel candidate Fusarium wilt resistance gene *FOC1* in *Brassica oleracea*

**DOI:** 10.1186/1471-2164-15-1094

**Published:** 2014-12-12

**Authors:** Honghao Lv, Zhiyuan Fang, Limei Yang, Yangyong Zhang, Qingbiao Wang, Yumei Liu, Mu Zhuang, Yuhong Yang, Bingyan Xie, Bo Liu, Jisheng Liu, Jungen Kang, Xiaowu Wang

**Affiliations:** Institute of Vegetables and Flowers, Chinese Academy of Agricultural Sciences, Key Laboratory of Biology and Genetic Improvement of Horticultural Crops, Ministry of Agriculture, 12#Zhongguancun Nandajie Street, Beijing, 100081 China; Beijing Vegetable Research Center, Beijing Academy of Agriculture and Forestry Sciences, Key Laboratory of Biology and Genetic Improvement of Horticultural Crops (North China), Ministry of Agriculture, 50# Zhanghua Street, Beijing, 100097 China

**Keywords:** *Brassica oleracea*, Fusarium wilt, Resistance gene, *FOC1*, Map-based cloning

## Abstract

**Background:**

Cabbage Fusarium wilt is a major disease worldwide that can cause severe yield loss in cabbage (*Brassica olerecea*). Although markers linked to the resistance gene *FOC1* have been identified, no candidate gene for it has been determined so far. In this study, we report the fine mapping and analysis of a candidate gene for *FOC1* using a double haploid (DH) population with 160 lines and a F_2_ population of 4000 individuals derived from the same parental lines.

**Results:**

We confirmed that the resistance to Fusarium wilt was controlled by a single dominant gene based on the resistance segregation ratio of the two populations. Using InDel primers designed from whole-genome re-sequencing data for the two parental lines (the resistant inbred-line 99–77 and the highly susceptible line 99–91) and the DH population, we mapped the resistance gene to a 382-kb genomic region on chromosome C06. Using the F_2_ population, we narrowed the region to an 84-kb interval that harbored ten genes, including four probable resistance genes (R genes): Bol037156, Bol037157, Bol037158 and Bol037161 according to the gene annotations from BRAD, the genomic database for *B. oleracea*. After correcting the model of the these genes, we re-predicted two R genes in the target region: re-Bol037156 and re-Bol0371578. The latter was excluded after we compared the two genes’ sequences between ten resistant materials and ten susceptible materials. For re-Bol037156, we found high identity among the sequences of the resistant lines, while among the susceptible lines, there were two types of InDels (a 1-bp insertion and a 10-bp deletion), each of which caused a frameshift and terminating mutation in the cDNA sequences. Further sequence analysis of the two InDel loci from 80 lines (40 resistant and 40 susceptible) also showed that all 40 R lines had no InDel mutation while 39 out of 40 S lines matched the two types of loci. Thus re-Bol037156 was identified as a likely candidate gene for *FOC1* in cabbage.

**Conclusions:**

This work may lay the foundation for marker-assisted selection as well as for further function analysis of the *FOC1* gene.

## Background

Cabbage (*Brassica oleracea* L. var. *capitata* L.) is one of the most cultivated vegetables worldwide. In 2010, more than two million hectares were estimated to be under production, with an average yield of 27.8 tonnes per hectare (FAOSTAT 2010). Cabbage Fusarium wilt (CFW) is a destructive fungal disease caused by *Fusarium oxysporum* f. sp. *conglutinans*, which for many years has caused severe losses to cabbage yields all over the world. CFW was first discovered in the USA in 1899, and then in Japan and several other countries in the following decades [1, 2]. In recent years, CFW was discovered in several provinces in China and has spread rapidly [3–5]. It is a typical soil-borne disease that is hard to control by traditional methods such as chemicals and crop rotation. The most effective control has been the use of resistant cabbage varieties [6–8]. Therefore, the development and use of new resources have become the main goals in current resistance breeding programs for cabbage.

It has been reported that two races in the formae speciale *conglutinans* could infect cabbage [9, 10]. Race 1 was found in several countries, and the inheritance of cabbage resistance to it was determined to be of the single dominant type [11, 12]. Race 2 was found only in the US and Russia, and the mode of inheritance of resistance to it remains unclear [13, 14].

Although CFW is a major disease for cabbage, not enough attention has been paid to it, and research on CFW is still at a preliminary stage. Several molecular markers have been developed for the *FOC* gene [15, 16], but, until now, no reports have focused on gene analysis or on functional studies of candidate genes. In a previous study, we located the resistance gene on chromosome C06 of cabbage, with two InDel markers flanking the gene at 1.2 and 0.6 cM [17]. Currently, the large scale re-sequencing of plant genomes has allowed for the application of insertion/deletion (InDel) and single nucleotide polymorphism (SNP) markers. In addition, the *B. oleracea* genome sequence has recently been published [18]. This has significantly advanced map-based cloning in cabbage, which was originally labor-intensive. InDel markers can be obtained easily, either using bioinformatic methods or from reliable visualization of alleles of different size [19].

The objectives of the current study were (i) to fine map the *FOC1* gene using one double haploid (DH) and one F_2_ population, and newly developed InDel markers designed from the whole-genome re-sequencing data of the two parental lines (the resistant inbred-line 99–77 and the highly susceptible line 99–91, and (ii) to analyze the genes that fell into the candidate genomic region, and finally identify the candidate gene.

## Results

### Resistance segregation of the DH and F_2_ populations

We obtained a total of 160 DH lines through microspore culture [20] and over 4000 F_2_ individuals derived from the same parental lines through hand pollination. Resistance segregation ratios in the DH and F_2_ populations were evaluated using the inoculation test. The result indicated that in the DH population, 68 lines showed immunity to Fusarium wilt while 92 were highly susceptible. The χ^2^ goodness-of-fit test indicated that the segregation ratio fitted a 1:1 ratio in the DH population. Of the 4000 individuals in the F_2_ population, 2964 plants were classified as resistant and 1012 were classified as susceptible; 24 individuals were excluded because of an unclear resistance performance. The χ^2^ goodness-of-fit test fitted a 3:1 Fusarium wilt resistance segregation ratio. These results indicated that the resistance in the two populations was controlled by a single dominant gene, which was consistent with the results from our prior study [5].

### Mapping of *FOC1*using the DH and F_2_populations

**Figure 1:**
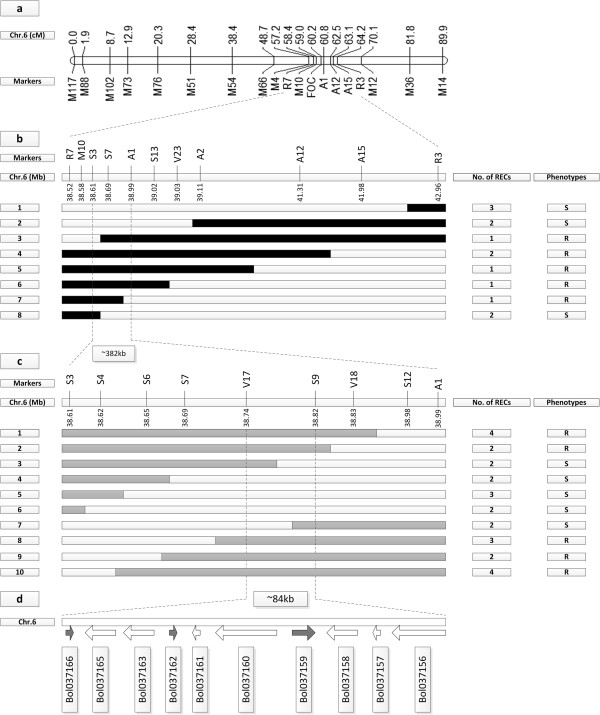
**Fine mapping of** ***FOC1*** **using the DH and F**_**2**_ **populations. (a)** The genetic map of 18 markers obtained from JoinMap 4.0. **(b)** Preliminary mapping of *FOC1* using the DH population. The *FOC1* gene was mapped to an interval of about 382 kb with S3 and A1 as the flanking markers according to the reference sequence of *B. oleracea*. **(c)** Fine mapping of *FOC1* using the F_2_ population with 3976 plants. The *FOC1* gene was delimited to an interval between V17 and S9, with an estimated length of 84 kb*.* **(d)** Ten genes were annotated between markers V17 and S9 based on the reference genome sequence. The genetic structure for each recombinant type is depicted as black rectangles for homozygous susceptible alleles, white for homozygous susceptible alleles and grey for heterozygous alleles, respectively. The relative positions of markers on C06 were determined according to the *B. oleracea* genome sequence.

In an earlier study, we located the candidate gene in a 1.8 cM genomic region on chromosome C06, with two InDel markers, M10 and A1, flanking *FOC1* at 1.2 cM and 0.6 cM respectively [17]. Based on this result, we designed additional InDel primers in this region to map the *FOC1* gene more precisely (Table 1). In addition, we obtained two pairs of simple sequence repeat (SSR) primers, R3 and R7, described in a previous study in which the candidate gene was mapped to a linkage group with two markers flanking the gene at 4.6 cM and 1.2 cM [16]. A total of 18 markers were anchored to the genetic map using JoinMap 4.0, which matched to the physical map according to the positions of the markers (Figure 1a). Finally, using these markers and the DH population, we mapped the *FOC1* gene to a 382-kb genomic region between the S3 and A1 markers (Figure 1b). Moreover, the SSR markers indicated that this was the same region as the region reported in the previous study (Figure 1b) [16].

**Table 1:** **Primers used for fine mapping of** ***FOC1***

Primer	Physical position on C06	Type	Tm	Sequences (5′-3′)
R7	38515717	SSR	58°C	TCACTCCTCTCGCAGATTCA//TGGAATCGCTTTAAGCAGATGC
M10	38577450	InDel	55°C	CACTTGCTCCAGTTTCTGTA//AACTATGGATAAAAGGCGTG
S3	38605567	InDel	55°C	TTTCGGTTGACAGAGAAAGT//AGGAGAGAATCAAAAGCTCC
S4	38610913	InDel	55°C	GGACTCACCTTTTTGTGTGT//AGATTACCCGTTTTCTCCTC
S6	38645784	InDel	55°C	GGACTCCTTTGATCCTCTCT//GAATGCAACCATGTAAACAG
S7	38685934	InDel	55°C	ACTTGACAGCTTTCCATCTG//TTTCTGCCCAGGTACTTAAA
V17	38743868	InDel	55°C	GCAGATAATAATCCACACGTC//TACCACCTTTTTCTGGCTTA
S9	38827074	InDel	55°C	CTGAAACTCGGGAATACATC//AAGCTTCCCACACTCTCTCT
V18	38828622	InDel	55°C	TACAAGCTCCTTGTGGATTT//CAATCCTTGGCTGTTTTAAG
S12	38986625	InDel	55°C	GCCCTCATTACGTTTAACAA//GAGTTGCAGCCTTCAAGTAT
A1	38988089	InDel	55°C	TGACATAACCACTAGGAGCA//GCAGAAGCTTTGATGAAGTT
S13	39018573	InDel	55°C	TGATGGACTGAAACCTAACC//AGGAGGATGAGGAAGAACC
V23	39029152	InDel	55°C	CTCTTTATTGCAACGACACA//TCAATACGTCACTCATCACG
A2	39107094	InDel	55°C	TGGTCTTTGGTCTCTTGTTT//AGCATTAACACTGACACCCT
A12	41310866	InDel	55°C	GCGAACCGACCCTAGTAT//CAAAGCCAAGAAGGTGTTTA
A15	41982164	InDel	55°C	GGTTTGAACCTCTGAAAATG//TGTGATTTCGGTTAGAGTCC
R3	42957515	SSR	58°C	CGCTATGGATAATGTGTTCA//ATTAACAGCGAGGATAGCAA

The flanking markers A1 and S3 obtained from the DH population were used to screen the F_2_ population for recombinant individuals, which were then evaluated for their resistance to Fusarium wilt. Next, more markers between A1 and S3 were used to genotype these individuals. As a result, the *FOC1* gene was narrowed to an 84-kb genomic region with V17 and S9 as the flanking InDel markers (Figure 1c). Both InDel markers had two recombinants with *FOC1*.

### Candidate gene analysis

From BRAD (http://brassicadb.org) [21], the genomic database for *B. oleracea* where the reference genome sequence for *B. oleracea* is now available [18], we obtained the reference genome sequence and found that there were ten annotated genes in the 84-kb genomic region (Figure 1d, Table 2). However, we noticed that there was no gene numbered Bol037164 in the database, and the prediction of the sequence between Bol037163 and Bol037165 showed that no gene existed here. The best hits of the ten annotated *B. oleracea* genes to the *Brassica rapa* and *Arabidopsis thaliana* genome are shown in Table 2. Annotation analysis of these genes from BRAD indicated that four of them were most likely related to disease resistance [22]: Bol037161 was predicted to have transcription factor activity; Bol037156, Bol037157 and Bol037158 contained the most commonly conserved domain of R proteins: leucine-rich repeat (LRR), toll-interleukin receptor (TIR) domain and LRR domain respectively (Table 2), based on the InterPro domain annotation and Gene Ontology annotation for *B. olerecea* and *A. thaliana* obtained from BRAD.

**Table 2:** **Annotation of the** ***B. oleracea*** **genes in the candidate region**

*Bra* genes ^a^	E-value	*Bol* genes ^b^	Gene position on C06 ^c^	*Bol* IPR annotation ^d^	*AT* ID ^e^	E-value	*AT* GO annotation ^f^
_	_	Bol037166	38746794-38747237	DNA polymerase	AT5G18880	5E-21	biological process unknown
_	_	Bol037165	38749215-38750398	Alpha/beta hydrolase	AT4G16690	8E-126	hydrolase activity
_	_	Bol037163	38753393-38754437	Ribosomal protein	AT4G16720	2E-107	ribosomal protein
_	_	Bol037162	38754883-38755278	Exostosin-like	AT4G16745	7E-68	exostosin family protein
_	_	**Bol037161**	**38795305-38795850**	**transcriptional factor**	**AT4G16750**	**2E-73**	**transcription factor**
_	_	Bol037160	38801162-38804053	oxygenase	AT4G16765	1E-117	oxidoreductase activity
Bra012690	0	Bol037159	38810947-38812479	Lipase	AT4G16820	0	lipase activity
Bra012689	0	**Bol037158**	**38813370-38815166**	**LRR**	**AT4G16950**	**2E-82**	**RPP5; nucleotide binding**
Bra012689	2E-90	**Bol037157**	**38816186-38816452**	**TIR**	**AT4G16890**	**1E-32**	**nucleotide binding**
Bra012688	0	**Bol037156**	**38822022-38824544**	**LRR**	**AT4G08450**	**2E-17**	**disease resistance protein**
Bra012687	0	Bol037155	38831452-38833049	Pollen allergen	AT4G17030	3E-117	cellulose and wall loosening

^a^ *B. rapa* homologous genes in the candidate region.

^b^Ten *B. oleracea* genes in the candidate region. The likely resistance genes and their information are indicated in bold.

^c^The physical position of the ten *B. oleracea* genes on chromosome C06.

^d^InterPro domain annotations for the ten *B. oleracea* genes obtained from BRAD.

^e^The best hits of the ten *B.oleracea* genes to *A.thaliana (AT).*

^f^GO annotations for the ten *Bol* to *AT* best-hit genes obtained from BRAD.

Primers were designed to amplify the full length of the four candidate genes in ten resistant inbred lines (including the resistant parental line) and ten susceptible inbred lines (including the susceptible parental line) (Table 3). However, no common sequence variation was found between the R_bulk and the S_bulk for all the four genes.

**Table 3:** **Primers used for cloning full length gene of four candidate genes**

Primers	Sequences (5′-3′)
Bol037156	GGTGACACTTCCTTCCTCCA//GCTCCATCGCCATCAAAGTT
Bol037157	TCGATTTTCCTCACCACCGA//TAAGCATCCCACCTGAGCAA
Bol037158	CTCAGATTGCTCAGGTGGGA//ACACACCCACATTGCGTTAC
Bol037161	GAAACTGTGTCCCTGCCATG//TGTGGCAGAGTTACATGGGT

**Figure 2:**
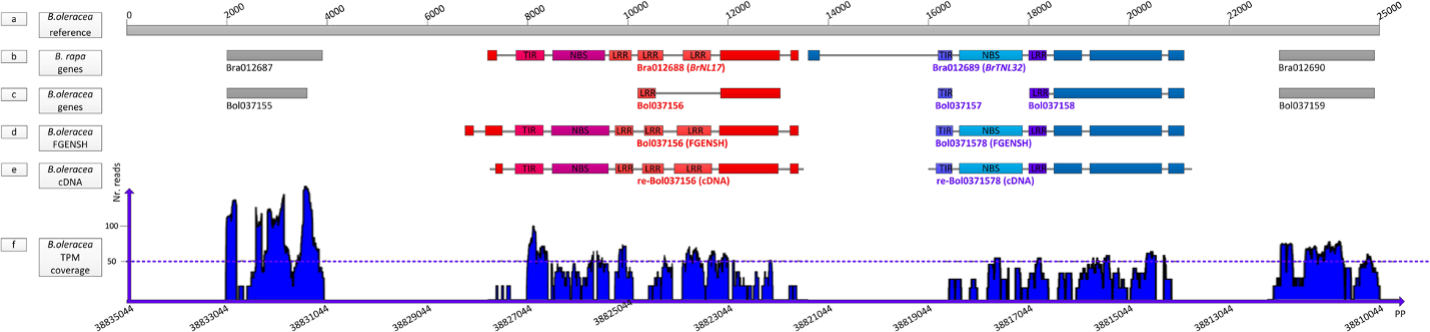
**Re-prediction of the genes in the candidate region. (a)** Reference genome sequence of *B. oleracea*; **(b)** Mapping results of *B. rapa* homologous genes in the candidate region of *B. oleracea*; The exons are depicted as rectangles. **(c)** The original positions of the *B. oleracea* genes in the candidate region; **(d)** Re-prediction of the *B. oleracea* genes in the candidate region using FGENSH program; **(e)** The mapping results of the cDNA sequence of the two re-predicted genes; **(f)** Reads mapping results of the *B. oleracea* transcriptome.

Nevertheless, all the three genes seemed too short with only one R protein domain based on a common sense of TIR-NBS-LRR or NBS-LRR type resistance gene (R gene) except Bol037161 as a transcription factor (Table 2) [22]. Thus comparative genomics analysis was made between *B. rapa* genes and *B. oleracea* ones in the target region (Figure 2a, b and c). The results showed that the homologous gene for Bol037156 in *B. rapa* was a putative TIR-NBS-LRR type R gene Bra012688, or *BrNL17* [GenBank: FJ842771.1] (http://blast.ncbi.nlm.nih.gov) [23], while the homologous gene for Bol037157 and Bol037158 was also a putative TIR-NBS-LRR type R gene Bra012689, or *BrTNL32* [GenBank: FJ842772.1] (Figure 2b and c). To further analyze these genes, the target genomic sequence of *B. olerecea* containing the three genes was submitted to the FGENSH program (http://linux1.softberry.com/berry.phtml) [24] to conduct gene prediction. Because the reference line was highly susceptible in our inoculation test which might affect the prediction result, the re-prediction program used the target sequence of the resistant parental line P_1_.

The results indicated that the re-predicted gene Bol037156, designated as re-Bol037156, was a putative TIR-NBS-LRR gene with high identity to Bra012688, while the other two genes Bol037157 and Bol037158 were re-predicted to be another putative TIR-NBS-LRR gene, named re-Bol0371578 which showed high identity to Bra012689 (Figure 2d).

The cDNA sequences showed high identity to the re-predicted genes’ sequences (Figure 2e). The *B. olerecea* transcriptome reads mapping results further confirmed the re-prediction result (Figure 2f). Thus, we obtained two re-predicted *B. oleracea* genes as the candidates, both of which were putative TIR-NBS-LRR type R genes.

**Figure 3:**
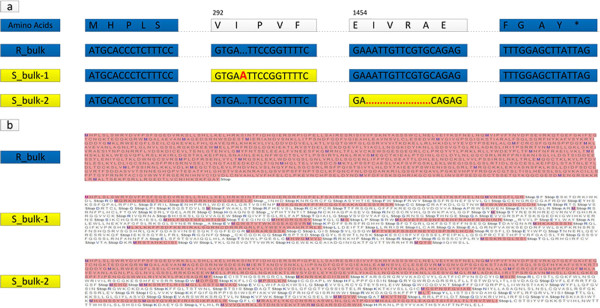
**Alignment of CDS sequences of candidate genes in ten R_bulk lines and ten S_bulk lines. (a)** Alignment of the CDS sequences. R_bulk: ten resistant lines, the coding sequences of ten R_bulk lines showed high identity and no InDels were found; S_bulk-1: all the susceptible lines except S1 and S3; one type of 1-bp insertion was found in S_bulk-1 to cause frame-shift and termination mutation; S_bulk-2: the susceptible lines S1 and S3; another type of 10-bp deletion was found to cause frameshift and termination mutation. **(b)** Frame-shift and termination mutation in the open reading frames of the susceptible lines caused by two types of InDels in the S_bulk lines.

To further identify which one was the most likely candidate gene for CFW resistance, primers were designed to obtain both full length DNA and cDNA of the two genes from ten resistant cabbage inbred lines including the resistant parent (R_bulk) and ten susceptible lines including the susceptible parent (S_bulk) in order to conduct sequence alignment and mutation loci analysis (Table 4). The results showed that no consistent variation was found between the R_bulk and the S_bulk for the gene re-Bol0371578. While for re-Bol037156, the cDNA from the ten R_bulk lines showed a high identity of 98.97% and no InDel was found. The cDNA from the ten S_bulk lines also showed high identity. When the R_bulk and S_bulk lines were compared, two types of InDels in the S_bulk were found; one was a 10-bp deletion in two sequences and the other was a 1-bp insertion in the other eight sequences (Figure 3a). Both InDels caused frameshifts in the open reading frames that resulted in terminating mutations in the susceptible lines (Figure 3b).

**Table 4:** **Primers used for cloning of re-Bol037156 and re-Bol0371578 in the R_bulk and S_bulk lines**

Primers	Sequences (5′-3′)
re-Bol037156 (DNA)	CCCCAGATTCACAGCATTCG//ATACGAGTGTGTCTTCGCCA
re-Bol037156 (cDNA)	ATCTTCACCACTCCAGTCA//ACTTAACTGGAATGAGCTAACCA
re-Bol0371578 (DNA)	TCGATTTTCCTCACCACCGA//ACCCAGCCCTAATGAAAGCT
re-Bol0371578 (cDNA)	TGAGTTGGTCCACGTACTTGA//AGGAGAAAGGGAAAGACGCA

Thus, the newly predicted gene re-Bol037156 was considered as a candidate gene for CFW resistance, with a total length of 6.4 kb, CDS length of 4.1 kb, eight exons and seven introns (Figure 2e). The candidate gene was predicted to encode a TIR-NBS-LRR type protein containing five conserved domains including one TIR domain, one NBS domain and three LRR domains according to a Pfam sequence search result (Figure 2e) (http://pfam.sanger.ac.uk/) [25].

### Validation of the *FOC1*gene

To verify re-Bol037156 as the candidate gene for cabbage Fusarium wilt resistance gene *FOC1,* primers were designed to obtain the two InDel loci sequence from 30 resistant cabbage inbred lines (R_bulk) and 30 susceptible lines (S_bulk) (Table 5). Together with the sequences of the former 20 lines, we conducted sequence alignment.

**Table 5:** **Primers used for two InDel loci analysis in 80 lines**

Primers	Sequences (5′-3′)
1-bp insertion	AATCACTCCTCAGCCATCTT//ATACCAGTTCCGAGAATCCT
10-bp deletion	AAGGAGGAGTGGATGGAG//GCTCTGATGTGTTGAAATAC

The sequences from the 40 R_bulk lines showed high identity and no InDel was found at the two loci. The 40 sequences from the ten S_bulk lines also showed high identity. When the R_bulk and S_bulk lines were compared, two types of InDels in the S_bulk were found as was described above: sequences of 29 lines matched the 1-bp insertion and sequences of other 10 lines matched the 10-bp deletion. However, sequence of one line (S12) in the S_bulk matched neither of the two types of InDels. As we didn’t obtain the full length cDNA of the gene in these lines, we concluded that there might be other types of variation that caused loss of gene function. The result of 80 lines’ sequences alignment further validated that re-Bol037156 was a most likely candidate for *FOC1*.

## Discussion

### Fusarium wilt and cabbage resistance breeding

CFW is a soil-borne disease that is hard to control by traditional physical or chemical methods. Therefore, CFW has spread quickly across China from Beijing in 2003 to the Hebei, Gansu, Shanxi, and Shaanxi provinces where it is now present. Thus, the application of resistant varieties is of great significance [6–8]. Indeed, their application has often been cited as a good example of using a plant’s own power to overcome fungal disease. Two factors account for this: firstly, the inheritance of cabbage resistance to Race 1 is single dominant [11, 12], as was also shown in our prior study [5], and this meant that the R gene could be used easily in the F_1_ progeny; and secondly, although two races in the forma speciale *conglutinans* can infect cabbage, only Race 1 is found worldwide, while Race 2 has been reported only in the USA and Russia [13, 14], indicating that the immutability of the pathogen over decades has also contributed to the successful use of the single dominant R gene. Because of the significance of this R gene, it is necessary to discover the mechanism by which this R gene overcomes CFW.

For many years, MAS has promoted the traditional breeding process. However, few studies have reported molecular markers for *FOC1*. Pu et al. [16] located the R gene on a linkage group, with two SSR markers flanking the gene at 4.6 and 1.2 cM respectively without chromosome information, and in a previous study, we located the R gene on chromosome C06, with two InDel markers flanking the gene at 1.2 and 0.6 cM [17]. In this study, we further refined the map and identified a candidate gene for *FOC1*. The results reported here will be useful for cabbage breeders because they may facilitate MAS in the breeding process.

### Map-based cloning of *FOC1*using InDel markers and two populations

Traditional molecular marker assays are usually labor-intensive and time-consuming, and the markers, like SSRs, that are used are frequently of low polymorphism and not reliable. Further, they often produce complicated stripes when the PCR products are isolated in the gels. In recent years, as more and more plant genome sequences become available including *B. oleracea* genome [18], InDel and SNP markers have become the markers of choice because they are generally obtained easily using bioinformatic method and their PCR products are more reliable and easier to detect. In this study, for example, we easily acquired 1000 pairs of InDel primers on one chromosome and although we used only a small number of them, the polymorphic primers accounted for 75% of all.

In this study, the InDel primers were designed based on the cabbage reference sequence and the whole-genome re-sequencing data for the parental lines. Therefore, the polymorphic primers accounted for a relative higher percentage. Also, genome-sequence-based InDel primers allow us to quickly design and add more markers to the target interval on the chromosome in order to narrow the mapping interval. As a result, we successfully mapped the R gene to chromosome C06 in a time-saving way based on the DH and F_2_ populations.

Most characterized R genes reside in clusters rather than being distributed equally on chromosomes [26]. Here also, the candidate region harbored two TIR-NBS-LRR genes: re-Bol037156 and re-Bol0371578.

### Plant resistance gene to disease caused by *Fusarium oxysporum*

As far as we know, more than 70 R genes have been cloned in the past decades [27]. The main categories of these genes include the coiled coil-nucleotide binding site-leucine rich repeat (CC-NB-LRR) class, the TIR-NBS-LRR class, the kinase class, the LRR kinase class and the extracellular LRRs [22]. The most significant TIR-NBS-LRR class R gene may be the tobacco *N* gene conferring resistance to tobacco mosaic virus [28]. For *N* gene, all the three conserved domains are indispensable for a normal function [29].

Until now, the Fusarium wilt R genes have been cloned in three plant species: Arabidopsis, tomato, and melon [30–32]. The function of the melon R gene, *FOM-2*, has not yet been established [31]. *RFO1* in Arabidopsis was reported to be a wall-associated kinase, which was neither a representative R gene nor race-specific [32]. On the other hand, the CFW R gene was race-specific and can be overcome by Race 2 [13]. In tomato, *I-2* was shown to be a typical R gene, with the encoded protein belonging to the CC-NBS-LRR type [30]. A low identity of 41% was found among the three cloned R genes, indicating that although all three were R genes to Fusarium wilt, different mechanisms may be involved. We also performed whole-genome blast searches with a cut-off value of < *E*-10, using the *RFO1, I-2* and *FOM-2* sequences as the queries. No matches to these sequences were found in the *B. oleracea* genome. Thus, we can infer that *FOC1* may be a novel R gene. The results of our study showed that the candidate gene for *FOC1* was a putative TIR-NBS-LRR type R gene. The cDNA sequences of the ten resistant and ten susceptible lines revealed two types of InDel mutations in the cDNA of the susceptible lines that resulted in frameshifts and translation termination. Further sequence analysis of the two InDel loci from 80 lines confirmed the two types of InDels. Thus, we inferred that the re-modeled gene re-Bol037156 was the most likely candidate gene for *FOC1.* However, further work is needed to verify the function of *FOC1* by transformation*.*

## Conclusions

In this study we fine mapped a novel cabbage resistance gene to Fusarium wilt using one DH population and one F_2_ population, analyzed the genes that fell into the candidate region and identified the re-modeled gene re-Bol037156, a putative TIR-NBS-LRR type R gene as the candidate for the cabbage Fusarium wilt resistance gene *FOC1*. This work may lay the foundation for marker-assisted selection in cabbage resistance breeding as well as for further function analysis of the *FOC1* gene.

## Methods

### Plant materials and pathogen strain

To generate F_1_ plants, the inbred-line 99–77 (P_1_, resistant parent) with immunity to CFW was crossed to the highly susceptible line 99–91 (P_2_, susceptible parent). An isolated microspore culture of F_1_ flower buds was adopted to obtain 160 DH lines [20]. At the same time, F_1_ plants were self-pollinated to generate about 4000 individual F_2_ plants. All the plant materials were provided by the Cabbage and Broccoli Research Group, Institute of Vegetables and Flowers (IVF), Chinese Academy of Agricultural Sciences (CAAS). The entire inoculation test was performed in the greenhouse of the Plant Protection Research Group at IVF, CAAS.

The strain FGL3-6 of *F. oxysporum* f. sp. *conglutinans* used in this study was isolated from diseased plants from Yanqing County, Beijing. This strain had been shown to be Race 1 [5].

### Evaluation of resistance to Fusarium wilt

For seedling cultivation, the cabbage seeds were sowed in a sterilized substrate (turf: vermiculite: soil = 1: 1: 2) and cultivated in a greenhouse until the third leaf stage.

For inoculum preparation, strain FGL3-6 of the pathogen, maintained on potato dextrose agar medium, was incubated in CM medium (complete medium with casein acids hydrolysate 10 g/l, casein enzyme hydrolysate 10 g/l, yeast extract 16 g/l, and lactose 20 g/l) on a rotary shaker (150 r/min, 26°C) for three days. Double-layer gauze was used to filter the mixture and make a conidium suspension. The concentration of the suspension was adjusted to 1 × 10^6^ conidia/ml for inoculation tests using a hemacytometer.

For the inoculation test, the root-dipping method was adopted to evaluate the resistance of the DH lines as well as the F_2_ individuals [9, 33, 34]. We picked the seedlings, dipped the roots in the conidial suspension for 15 minutes, and then planted them in plastic pots (diameter × height = 9 cm × 9 cm) with sterilized substrate (turf: vermiculite = 1: 2). The seedlings were cultivated in a greenhouse with temperatures of 27–29°C in the day and 23–25°C at night. The resistance levels of the plants were evaluated after 10 days.

In a previous study, we found that Fusarium resistance in the populations used in our study was controlled by a single dominant gene. Therefore, for disease rating in the present study, a simple two-grade disease rating standard was adopted to classify the DH lines and F_2_ individuals as resistant or susceptible. For the DH population, a randomized trial design was adopted with three replications and each replication consisted of 10 plants. Lines or individuals for which the resistance status was unclear were excluded from further analysis. In the inoculation test, the numbers of resistant and susceptible plants in the DH and F_2_ populations were counted. Then, χ^2^ goodness-of-fit tests were applied to determine whether the segregation ratios fitted a 1:1 ratio in the DH population and fitted a 3:1 ratio in the F_2_ population at a significance level of 0.05.

### DNA extraction

The cetyl trimethyl ammonium bromide (CTAB) method with minor modifications was used to extract total genomic DNA from the young leaves of 20-day-old plants [35]. The concentration of the DNA samples was determined using a NanoDrop ND-100 spectrophotometer (Thermo Fisher Scientific Inc., Wilmington, DE, USA), and then diluted to a working concentration of 40 ng/μl for PCR analysis.

### Molecular maker assay

The reference sequence of *B. olerecea* was retrieved from the Brassica database (BRAD) (http://brassicadb.org) [21] and used for InDel primer design. The DNA of the parental lines was submitted to whole-genome re-sequencing using the sequencing-by-synthesis method. A total of 7.2 Gb and 7.1 Gb Illumina pair-end reads were generated for the P_1_ and P_2_ parental lines respectively. The reference sequence was used as a ‘bridge’ to detect sequence polymorphisms between the parental lines [36]. To avoid false detection of polymorphisms, multiple-hit reads were filtered out from the dataset and only single-hit reads were used to design primers. In a prior study, we located the resistance gene to a 1.8 cM genomic region [17]. To map the gene more precisely, additional InDel primers were designed on chromosome C06. For all the primers, the generating products was constrained to be 100-200 bp. The length of primer pairs was limited to 19–22 bp. The Tm value was restricted to be 54–56°C.

The PCR conditions and contents were as follows: each 20 μl PCR reaction mixture contained 2 μl PCR buffer (10×, Mg^2+^ included), 1.6 μl dNTP (2.5 mM each), 0.4 μl Taq DNA polymerase (2.5 U/μl), 5 μl DNA template (40 ng/μl), 0.6 μl each of forward and reverse primers (10 μM), and 9.8 μl ddH_2_O. The reaction mixture was incubated in a GeneAmp PCR system 9700 (Applied Biosystems, CA, US) and the PCR profile was as follows: initial 5 min at 94°C, then 35 cycles, each with 30 s DNA denaturation at 94°C, 30 s annealing at 55°C and 45 s extension at 72°C, and a final extension of 7 min at 72°C. The PCR products were separated on an 8% (w/v) polyacrylamide gel at 160 V for 1.5 h and stained with silver nitrate [37].

### Statistical analysis and gene mapping

In the DH population, for each pair of primers, the allele of an individual that was the same as the corresponding allele in P_1_ (99–77) was scored as ‘a’, while the allele that was the same as the corresponding allele in P_2_ (99–91) was scored as ‘b’. The data was analyzed with JoinMap 4.0 using a minimum logarithm of odds (LOD) score of 4.0 [38]. The Kosambi function was used to convert the recombinant value to genetic distance [39]. In this step, the gene was preliminarily mapped to an interval with two flanking markers.

The flanking markers obtained from the DH population were used to screen the F_2_ population for recombinants. The recombinants were further genotyped using the markers that mapped between the flanking ones. Except for the ‘a’ and ‘b’ alleles, individuals that had both the alleles from the two parents were scored as ‘h’. In this step, we obtained a narrowed candidate interval with new flanking markers.

To identify the probable genes associated with disease resistance, genes that fell into the candidate interval were analyzed based on the annotations for the *B. oleracea* reference chromosomes from BRAD.

### Analysis of the candidate gene

To identify which one was the candidate gene, primers were designed and used to amplify the genomic DNA and cDNA of ten resistant cabbage inbred-lines (R_bulk) including the resistant parent P_1_ and ten susceptible lines (S_bulk) including the susceptible parent P_2_. The obtained sequences were aligned to detect common differences between the R_bulk and S_bulk lines. Comparative genomics analysis between *B. rapa* and *B. oleracea* in the target region was performed in order to help identify candidate genes. RT-PCR and transcriptome reads (RNA sequencing was adopted to obtain the transcriptome using RNA extracted from the roots of the seedling six days after inoculation) mapping were also conducted to help confirm the candidate gene for *FOC1*.

### Validation of the candidate gene

To further validate the candidate gene for *FOC1*, we chose 40 different highly resistant cabbage inbred-lines including the former ten resistant lines to make the R_bulk and 40 highly susceptible lines including the former ten susceptible lines to make the S_bulk. Primers were designed to amplify the mutation loci of the candidate gene in these 80 cabbage inbred lines. The obtained sequences were aligned to detect common variation between the R_bulk and S_bulk lines.

### Availability of supporting data

The reference genome sequence of *B. oleracea* was retrieved from BRAD, the genomic database for *B. oleracea* (available at http://brassicadb.org). As one of the data owners, we confirm that we have full permission to use the genome sequence of *Brassica oleracea*, including downloading and using it for the purposes of this study (i.e. candidate gene identification and characterization). The use of the reference genome for these purposes was performed with the agreement of all the data owners.

## Abbreviations

*FOC1*: Resistance gene to Race 1 of *Fusarium oxysporum* f. sp. *conglutinans*
CFW: Cabbage Fusarium wilt
DH: Double haploid
CDS: Protein coding sequence
InDel: Insertion/deletion
MAS: Marker-assisted selection
SNP: Single nucleotide polymorphism
PCR: Polymerase chain reaction
R gene: Resistant gene
S gene: Susceptible gene
SSR: Simple sequence repeat
NBS: Nucleotide binding site
TIR: Toll-interleukin receptor
LRR: Leucine-rich repeat
RT-PCR: Reverse transcription-PCR
CC: Coiled coil.
